# Clarithromycin use for adjunct surgical prophylaxis before non-elective cesarean deliveries to adapt to azithromycin shortages in COVID-19 pandemic

**DOI:** 10.1371/journal.pone.0244266

**Published:** 2020-12-21

**Authors:** Daniel Martingano, Ashley Nguyen, Claudia Nkeih, Shailini Singh, Antonina Mitrofanova

**Affiliations:** 1 Department of Obstetrics & Gynecology, St. John’s Episcopal Hospital, Far Rockaway, NY, United States of America; 2 Department of Biomedical and Health Informatics, Rutgers University School of Health Professions, Newark, NJ, United States of America; 3 Department of Obstetrics & Gynecology, Trinitas Regional Medical Center, Elizabeth, NJ, United States of America; 4 Division of Maternal-Fetal Medicine, Department of Obstetrics & Gynecology, Newark Beth Israel Medical Center, Newark, NJ, United States of America; 5 Department of Obstetrics & Gynecology, Rutgers Robert Wood Johnson Medical School, New Brunswick, NJ, United States of America; 6 Rutgers Cancer Institute of New Jersey, New Brunswick, NJ, United States of America; University of Insubria, ITALY

## Abstract

**Objective:**

This study aimed to evaluate safety and effectiveness of clarithromycin as adjunctive antibiotic prophylaxis for patients undergoing non-elective cesarean delivery in comparison with no macrolides, to adapt to azithromycin shortages in COVID-19 pandemic.

**Study design:**

We conducted a multi-center, prospective observational cohort study from March 23, 2020 through June 1, 2020. We followed all women receiving either clarithromycin or no macrolide antibiotic for adjunct surgical prophylaxis for non-elective cesarean deliveries. The primary outcome was development of postpartum endometritis. Secondary outcomes included meconium-stained amniotic fluid at time of cesarean delivery, neonatal sepsis, neonatal intensive care unit admission, and neonatal acute respiratory distress syndrome. All patients in this study were tested for SARS-CoV-2 infection and resulted negative.

**Results:**

This study included 240 patients, with 133 patients receiving clarithromycin and 107 patients receiving no adjunct macrolide prophylaxis. Patients receiving clarithromycin were noted to have significantly lower rates of postpartum endometritis as compared to those who did not receive adjunct prophylaxis (4.5% versus 11.2%, *p* = 0.025). In crude (unadjusted) analysis, a significantly lower risk of developing endometritis was noted in the clarithromycin group as compared to the control group (66% decreased risk, 95% CI 0.12 to 0.95, *p* = 0.040). When adjusted for perceived confounders, a significant difference was again noted (67% decreased risk, 95% CI 0.11 to 0.97, *p* = 0.034). Stratified analysis of significantly different demographic factors including Black race, BMI, and age was performed. A significantly decreased risk of development of endometritis when taking clarithromycin versus no adjunct macrolide was noted for Black race women in crude and adjusted models (crude: 87% decreased risk, 95% CI 0.08 to 0.83, *p* = 0.032; adjusted: 91% decreased risk, 95% CI 0.06 to 0.79, *p* = 0.026). This was also noted for women aged 18–29 years in crude and adjusted models (crude: model, 79% decreased risk, 95% CI 0.06 to 0.80, *p* = 0.014; adjusted model: 75% decreased risk, 95% CI 0.06 to 0.94, *p =* 0.028). All other stratified analyses did not yield significant differences in endometritis risk.

**Conclusion:**

Our study suggests that administration of clarithromycin for adjunctive surgical prophylaxis for non-elective cesarean deliveries may be a safe option that may provide suitable endometritis prophylaxis in cases where azithromycin is unavailable, as was the case during the start of COVID-19 pandemic, most especially for Black race women and women ages 18–29 years.

## Introduction

The United States is presently involved in a worsening pandemic due to the outbreak and spread of a novel coronavirus called severe acute respiratory syndrome coronavirus 2 (SARS-CoV-2) causing the disease known as COVID-19. This was identified first in Wuhan, China in late 2019 with the first case in the United States in Washington State in January 2020 [1, 2]. Information about SARS-CoV-2 is evolving rapidly, and interim guidance by multiple organizations is constantly being updated and expanded [3–6]. Given the acute nature of this pandemic, a wide variety of medical resources are being rapidly consumed resulting in an insult to the global supply chain and nationwide shortages of medications, personal protective equipment, and many other essential items [7, 8].

At the start of this specific research study, many institutions were utilizing azithromycin with or without hydroxychloroquine [3] as medical treatment for confirmed cases of COVID-19, resulting in national shortages of the azithromycin [7, 8] limiting its use for previously routine treatments, including adjunct surgical antibiotic prophylaxis for non-elective cesarean deliveries. However, as this study continued and more evidence was gathered globally on the effectiveness of this treatment approach, it was determined that there was no benefit for the use of azithromycin with or without hydroxychloroquine in the treatment of COVID-19, which ceased its use across institutions [9–14]. Because azithromycin was limited or unavailable due to demands for COVID-19 at the start of this study, this study aimed to evaluate the safety and effectiveness of using an alternative treatment with clarithromycin as adjunctive antibiotic prophylaxis for patients undergoing non-elective cesarean delivery, in comparison with no adjunctive prophylaxis.

Azithromycin and clarithromycin are structurally-similar derivatives of the older macrolide antibiotic erythromycin. While it may be a possible consideration to substitute erythromycin for azithromycin, many hospital pharmacies do not readily have erythromycin either intravenous or oral on their formularies [15]. Furthermore, the structural modifications made to erythromycin significantly broadened the spectrum of antibacterial activity of its derivatives, making them a more effective option in the treatment of a variety of infections, including community-acquired respiratory tract infections, gastrointestinal infections, and sexually transmitted chlamydial infections [16, 17].

Based on a pivotal study performed by Tita et al [18], azithromycin is currently utilized as adjunct antibiotic prophylaxis for non-elective cesarean deliveries. This study was a multi-center, randomized controlled trial evaluating the benefits of 500 mg of intravenous azithromycin in addition to a standard antibiotic prophylaxis regimen in 2,013 women undergoing non-elective cesarean deliveries. Women who received adjunctive azithromycin had significant reductions in the endometritis as well as primary composite outcome of endometritis, wound infection, or other infections together. There was no significant difference in the neonatal composite outcome that included death and serious neonatal complications. Given the favorable outcomes with adjunct azithromycin administration over placebo, it is a widely adopted practice across the United States to administer azithromycin as part of standard antibiotic prophylaxis for women undergoing non-elective cesarean deliveries, which is supported by the American College of Obstetrics & Gynecology [19].

Given shortages of azithromycin during the start of the COVID-19 pandemic and that azithromycin and clarithromycin are in the same class of antibiotics, this study aimed to evaluate the safety and effectiveness of using clarithromycin as adjunctive antibiotic prophylaxis for patients undergoing non-elective cesarean delivery in comparison to no adjunctive macrolide in situations where azithromycin is unavailable.

## Materials and methods

We conducted a multi-center, prospective observational cohort study from March 23, 2020 through June 1, 2020. The centers included in this study were two secondary care facilities located in Elizabeth, New Jersey and Far Rockaway, New York, as well as a tertiary care center located in Newark, New Jersey. All sites were located in culturally and economically diverse areas. The study’s conclusion date was determined to be June 1, 2020 because at that time, consensus was reached nationally that azithromycin with or without hydroxychloroquine was not an effective treatment for COVID-19 and supply of azithromycin were restored across study sites.

All patients undergoing a non-elective cesarean delivery during the study period were included. Included patients were followed from time of admission through discharge from inpatient obstetrical and postoperative care. Data was obtained prospectively through institutional electronic medical records. Patients were excluded if they were diagnosed with an intrapartum infection (including SARS-CoV-2, intraamniotic infection/inflammation, pyelonephritis, etc.), if antibiotics were administered in the postpartum period in cases of non-gynecologic infections (such as pneumonia, cellulitis, etc.) apart from the development of postpartum endometritis, had an allergy or contraindication to clarithromycin, were less than 37 weeks gestational age, experienced vomiting following administration of clarithromycin, or had missing follow up information or incomplete data. All patients in this study were tested for SARS-CoV-2 on admission and had a negative result.

All patients received standard surgical prophylaxis for cesarean delivery, which across all sites included IV cefazolin for which the dose ranging between 1–3 grams was dependent on patient body-mass index (BMI), surgical operating times, and surgical blood loss. As all cesarean deliveries have an inherent risk for increased operating time and surgical complications such as acute blood loss, bladder injury, etc. [20–23], adjustments to cefazolin dosing for surgical prophylaxis were made as needed.

In patients receiving clarithromycin for adjunct prophylaxis, 500mg was administered orally 30 minutes before skin incision. Decision on whether or not to administer clarithromycin was based upon specific hospital protocols applicable to all patients uniformly. Clarithromycin was offered to all patients requiring non-elective cesarean deliveries. Patients did not receive clarithromycin as adjunct surgical prophylaxis if time between decision for surgery and actual skin incision was less than 30 minutes, patient could not tolerate oral medications, or intervention was refused by provider. Patients who did not receive clarithromycin for any of the aforementioned reasons were included in the control group for this study.

The primary outcome was development of postpartum endometritis. Secondary outcomes included meconium-stained amniotic fluid at time of cesarean delivery, neonatal sepsis (both suspected and confirmed), neonatal intensive care unit admission, and neonatal acute respiratory distress syndrome. This study at all participating sites was approved by the institutional review board at St. John’s Episcopal Hospital.

### Statistical analysis

To account for unequal variance between treatment groups, Welch two-sample two-tailed *t*-test [24] was used to evaluate differences between continuous variables as appropriate. To evaluate differences between frequencies of binary variables between the treatment groups, we employed _*X*_^*2*^ test [25] and Fisher’s exact test [26] for comparing contingency tables as appropriate.

Relative risks (crude and adjusted) of binary pregnancy outcomes between treatment groups were calculated using “modified Poisson regression”. According to Zhou [27], “modified Poisson regression” is defined as Poisson regression using robust error variance called sandwich estimation. Adjusted models were controlled for perceived confounding factors including age, BMI, race, group-β *streptococcus* status, parity, gestational age, hospital site, induction of labor, and indication for cesarean delivery. Stratified analysis for significantly different demographic factors including Black race, BMI, and age was also performed. Likelihood ratio test was used to evaluate significance level for relative risk in crude and adjusted models. The power of the study was calculated post-hoc whereby it was determined that given our final sample size (n = 240), the power for continuous outcomes was 97% and 81% for binary outcomes to detect a 50% difference (α of 0.05 for a two-tailed test.) Statistical significance was defined by *p* values <0.05. All statistics were performed using R version 3.4.0 [28].

## Results and discussion

**Fig 1:**
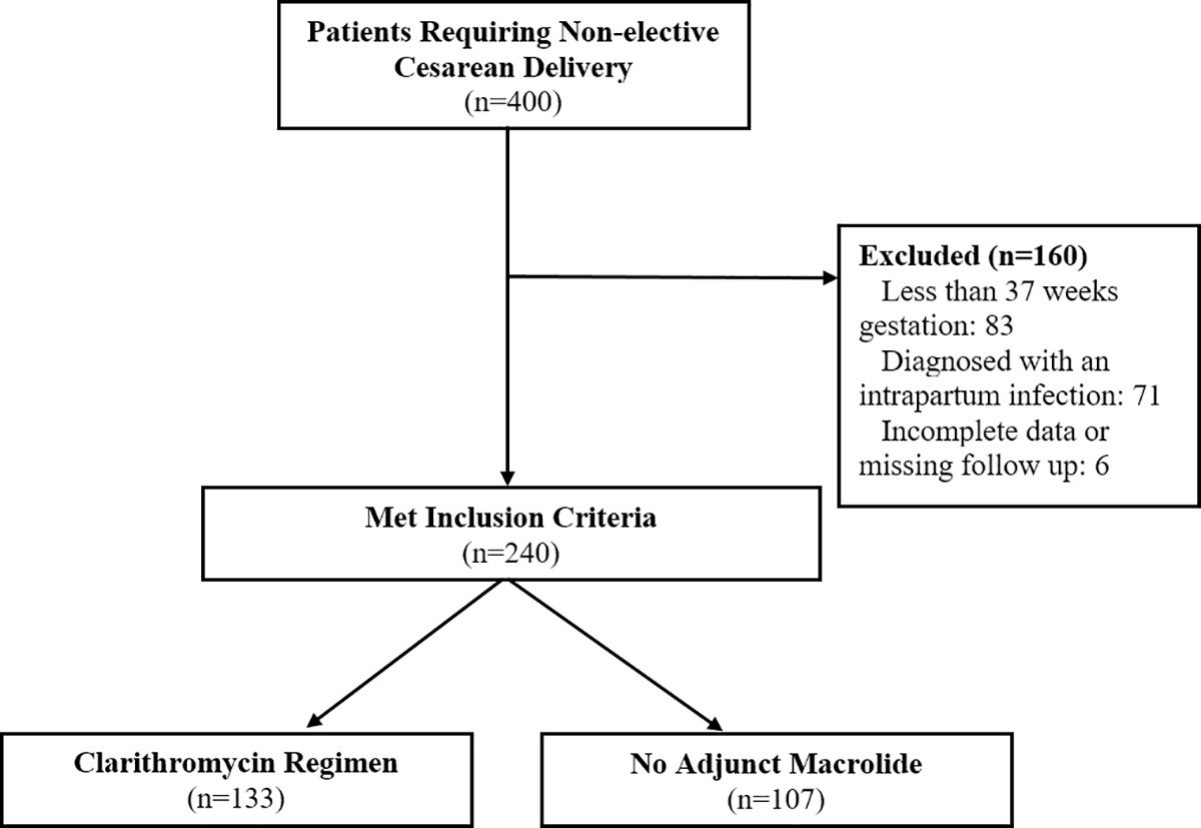
Study enrollment. A total of 400 patients were identified as requiring non-elective cesarean deliveries. 83 patients were excluded because they had pregnancies less than 37 weeks gestation at time of surgery. 71 patients were excluded because they received antibiotics apart from standard surgical prophylaxis due to intrapartum infection (43 patients intraamniotic infection/inflammation, 13 pyelonephritis, and 15 unspecified infections). 6 patients were excluded because of missing follow up information or incomplete data. No patients met any of the other exclusionary criteria.

Following application of the study’s inclusion/exclusion criteria, our study enrolled 240 patients (Fig 1), with 133 patients receiving clarithromycin and 107 patients receiving no adjunct macrolide prophylaxis (referred to as control group). Demographic information is presented in Table 1. BMI and patient age were noted to be significantly different between treatment groups, with the clarithromycin group having a lower maternal age (29.4 versus 31.3 years, *p* = 0.027, Welch two-sample two-tailed *t*-test) and lower BMI (31.7 versus 34.2 kg/m^2^, *p* = 0.038, Welch two-sample two-tailed *t*-test). Significant differences in the races of women between treatment groups were also noted, with the clarithromycin group having a higher percentage of Black race women (36.1% versus 22.4%, *p* = 0.022, _*X*_^2^ test) and lower percentage of White race women (41.3% versus 54.2%, *p* = 0.047, _*X*_^2^ test) while there were no differences in Asian or Hispanic race. All other demographic information was not significantly different.

**Table 1:** Maternal demographics.

Characteristic	Clarithromycin Group n = 133	No Macrolide Group n = 107	*p*
Maternal age (years)	29.4 ± 6.1 (19–41)	31.3 ± 6.8 (13–41)	0.027*
Advanced Maternal Age	33 (24.8)	39 (36.4)	0.051^ᵟ^
Gestational Age at Diagnosis	38.9 ± 1.3 (35–41)	39.1 ± 1.5 (37–41)	0.497*
Nulliparous	58 (43.6)	48 (44.9)	0.846^ᵟ^
BMI (kg/m^2^)	31.7 ± 9.4 (18–52)	34.2 ± 9.2 (18–50)	0.038*
Group β-streptococcus positive	32 (24.1)	20 (18.7)	0.316^ᵟ^
Hypertensive Disorders in Pregnancy	30 (22.6)	20 (18.7)	0.464^ᵟ^
Previous Cesarean Delivery	18 (13.5)	20 (18.7)	0.277^ᵟ^
> 2 Prior Cesarean Deliveries	10 (7.5)	14 (13.1)	0.153^ᵟ^
Induction of Labor	56 (42.1)	42 (38.3)	0.552^ᵟ^
Race			
White	55 (41.3)	58 (54.2)	0.047^ᵟ^
Asian	16 (12.1)	8 (7.5)	0.243^ᵟ^
Hispanic	14 (10.5)	17 (15.9)	0.218^ᵟ^
Black	48 (36.1)	24 (22.4)	0.022^ᵟ^
Indication for Cesarean			
Rupture of Membranes with Prior Cesarean (Not TOLAC Candidate)	18 (13.5)	20 (18.7)	0.277^ᵟ^
Failure to Progress	52 (39.1)	28 (26.2)	0.060^ᵟ^
Non-reassuring Fetal Heart Rate	62 (46.6)	58 (54.2)	0.243^ᵟ^
Other	1 (0.8)	1 (0.9)	N/A

Data are presented as mean ± standard deviation (range) or n (%)

ᵟ Statistics performed using _X_^2^ test

^F^ Statistics performed using Fischer’s Exact Test

*Statistics performed using Welch two-sample two-tailed t-test

Rates of binary pregnancy outcomes are shown in Table 2. Patients receiving clarithromycin were noted to have significantly lower rates of postpartum endometritis as compared to the control group (4.5% versus 11.2%, *p* = 0.025, _*X*_^2^ test). All other outcomes were not noted to be significantly different. No patients who received clarithromycin experienced severe side-effects including hepatotoxiticy, nephrotoxicity, cardiac arrhythmias, or significant gastrointestinal symptoms.

**Table 2:** Maternal and neonatal outcomes.

Outcome	Clarithromycin Group n = 133	Control Group n = 107	*p*
Maternal			
Postpartum Endometritis	6 (4.5)	12 (11.2)	0.025^δ^
Meconium Stained Amniotic Fluid (at time of cesarean)	22 (16.5)	14 (13.1)	0.456^δ^
Neonatal			
Intensive Care Unit Admission	10 (7.5)	5 (4.7)	0.365^δ^
ARDS	2 (1.5)	3 (2.8)	0.658^F^
Neonatal Sepsis (Suspected)	3 (2.3)	4 (3.7)	0.703^F^
Neonatal Sepsis (Confirmed)	0 (0)	0 (0)	N/A

Data are presented as mean ± standard deviation (range) or n (%)

^δ^ Statistics performed using _X_^2^ test

^F^ Statistics performed using Fischer’s Exact Test

*Statistics performed using Welch two-sample two-tailed t-test

Crude and adjusted risks ratios for pregnancy outcomes are shown in Table 3. In crude (unadjusted) analysis, a significantly lower risk of developing endometritis was noted in the clarithromycin group as compared to the control group (66% decreased risk, 95% CI 0.12 to 0.95, *p* = 0.040, Likelihood ratio test). When adjusted for perceived confounders, a significant difference was again noted (67% decreased risk, 95% CI 0.11 to 0.97, *p* = 0.034, Likelihood ratio test). All other outcomes were not noted to be significantly different.

**Table 3:** Crude and adjusted risk ratios for maternal and neonatal outcomes.

	Crude			Adjusted*		
	RR	95% CI	*p*^*+*^	RR	95% CI	*p*^*+*^
Postpartum Endometritis	0.34	0.12 to 0.95	0.040	0.33	0.11 to 0.97	0.034
Meconium-Stained Amniotic Fluid	1.26	0.65 to 2.47	0.493	1.17	0.58 to 2.37	0.662
Neonatal ICU Admission	1.61	0.55 to 4.71	0.385	1.67	0.48 to 5.84	0.416
Neonatal Sepsis (Suspected)	0.60	0.14 to 2.70	0.508	0.52	0.08 to 3.32	0.484

^+^*p* values calculated using Likelihood Ratio Test

Estimates are calculated via modified Poisson generalized linear models.

RR = risk ratio, CI = confidence interval.

Given significant differences in BMI, age, and race between treatment groups, we performed stratified analyses for crude and adjusted risk ratios for the development of postpartum endometritis, shown in Table 4. A significantly decreased risk of development of endometritis when taking clarithromycin versus control was noted for Black race women in crude and adjusted models (crude: 87% decreased risk, 95% CI 0.08 to 0.83, *p* = 0.032; adjusted: 91% decreased risk, 95% CI 0.06 to 0.79, *p* = 0.026, Likelihood ratio test). This was also noted for women aged 18–29 years in crude and adjusted models (crude: 79% decreased risk, 95% CI 0.06 to 0.80, *p* = 0.014; adjusted: 75% decreased risk, 95% CI 0.06 to 0.94, *p =* 0.038, Likelihood ratio test). All other stratified analyses did not yield significant differences in endometritis risk.

**Table 4:** Stratified crude and adjusted risk ratios for development of postpartum endometritis for patients receiving clarithromycin.

	Crude			Adjusted*		
	RR	95% CI	*p*^*+*^	RR	95% CI	*p*^*+*^
All Patients (n = 240)	0.34	0.12 to 0.95	0.040	0.35	0.11 to 0.97	0.034
Analysis By Race						
Black Race (n = 72)	0.13	0.08 to 0.83	0.032	0.09	0.06 to 0.79	0.026
White Race (n = 113)	0.70	0.20 to 2.49	0.582	0.71	0.18 to 2.75	0.618
Analysis by Age						
Ages 18–29 years (n = 116)	0.21	0.06 to 0.80	0.014	0.25	0.06 to 0.94	0.028
Ages >30 years (n = 124)	0.66	0.16 to 2.77	0.571	0.82	0.18 to 3.67	0.797
Analysis by BMI						
Class I-II Obese, BMI 30–39.9 (n = 77)	0.49	0.12 to 1.95	0.309	0.48	0.11 to 2.07	0.310
Class III Obese, BMI >40 (n = 66)	0.56	0.10 to 3.08	0.509	1.00	0.18 to 5.48	0.993

^+^*p* values calculated using Likelihood Ratio Test

Estimates are calculated via modified Poisson generalized linear models.

RR = risk ratio, CI = confidence interval.

### Extended analysis for Black race and age

Given that significant decreases in risk were noted for Black race women and women aged 18–29 years, respectively, we performed extended analysis for patients who were both Black race and aged 18–29 years, n = 40, where a significant decreased risk was noted with *p* = 0.006 in the adjusted model. A perfect treatment outcome (i.e. 0 patients of Black race and 18–29 years developing endometritis when receiving clarithromycin as compared to 3 patients that developed endometritis in the control group) was noted. These results suggests an extended benefit of postpartum endometritis prophylaxis for not just Black race women and women ages 18–29 respectively, but also those Black race women within that age range specifically, despite the small sample size. Overall, we found significantly lower rates of postpartum endometritis in the clarithromycin group as well as decreased risk for the development of postpartum endometritis in both crude and adjusted models. This suggests that there may be a benefit in reducing this complication following cesarean delivery, most especially for Black race women and women ages 18–29.

### Limitations

Due to the burden of the COVID-19 pandemic at the time this study was designed, associated limitations of resources precluded its organization as a randomized, controlled trial (RCT). Our study was most feasibly conducted as a prospective, observational study with a relatively small sample size, and thus cannot be directly compared to the aforementioned landmark RCT by Tita et al [18] which demonstrated azithromycin’s benefit following non-elective cesarean deliveries. Because our study design is not an RCT, our findings may be affected by possible biases despite our methods for controlling for confounding factors.

For example, because this study is not randomized, there is a possibility for selection bias. We have attempted to mitigate selection bias in our study in a couple of ways. While decision whether to give clarithromycin was based upon specific hospital protocols applicable to all patients uniformly, the study’s policy overall was to include all patients requiring a non-elective cesarean delivery in the clarithromycin group. Patients did not receive clarithromycin as adjunct surgical prophylaxis mainly if there was a systems issue precluding its timely administration rather than a selection to not administer the medication. Given these inherent limitations, our adjusted analysis was performed to control for many factors including hospital site and indication for cesarean delivery, both of which are likely sources of selection bias. These measures, however, are not a substitute for a randomized-controlled study design, and this remains a limitation of our study.

Furthermore, our study conclusions are limited in that we only used postpartum endometritis as our primary treatment outcome rather than including also surgical site infection (SSI) and other infectious morbidity as Tita et al [16] did. Our study only followed patients while they were admitted for inpatient obstetric and postoperative care. Of note, no patients tested positive for SARS-CoV-2 on admission or throughout their inpatient postpartum stay. We did not follow these patients for their outpatient visits when SSI would be identified or track if they returned with SSIs requiring more extensive treatment. This was mainly due to lack of resources to perform this follow up surveillance during the burden of COVID-19 across sites rather than lack of scientific inquiry. Tita et al [16] found a reduction in rates of endometritis alone in patients receiving adjunct antibiotic prophylaxis with azithromycin as compared to placebo (3.8% vs. 6.1%, *p* = 0.02), which is a similar outcome to what was observed in our study where patients receiving clarithromycin were noted to have significantly lower rates of endometritis as compared to control (4.5% versus 11.2%, *p* = 0.025). Our study, however, did not collect data on SSI, which remains a limitation of our study.

Finally, the motivation for the study in its conception was predicated on the fact that azithromycin was not available for adjunct antibiotic prophylaxis due to shortages of the medicine when it was repurposed as a treatment for COVID-19. Thus, we did not have an opportunity to have a comparison group for patients receiving the standard regimen with azithromycin. This remains a limitation of our study.

### Azithromycin and COVID-19

At the start of our research study, both our study’s institutions as well as centers nationally were utilizing azithromycin with or without hydroxychloroquine as medical treatment for confirmed cases of COVID-19. This widespread repurposing of azithromycin as a COVID-19 treatment resulted in national shortages of the azithromycin [7, 8]. While published reports on this shortage of azithromycin due to COVID-19 utilization specifically in the United States are sparse, there are two recent studies [8, 9] that highlight the shortage’s timeline and extent. For example, a report by Badreldin and Attalah [7] describes the global drug shortages due to COVID-19 and the impact it had on limiting azithromycin for usual treatments secondary to its repurposing as a COVID-19 treatment. Per the authors, this shortage was caused mainly by the resultant stockpiling and hoarding behaviors of azithromycin in response. Another recent study by Catillon et all [8] gathered data regarding reporting about medicines that were repurposed as COVID-19 treatments and noted to be in shortage by the United States’ Food and Drug Administration (FDA). Reporting regarding shortage of azithromycin specifically was noted to first rise around March 15, 2020 and peak around April 10, 2020. The reported shortage then decreased to an end around July 9, 2020. These dates are significant in that they coincide with our study’s starting and ending dates, highlighting that these trends noted nationally are an accurate reflection of what our institutions experienced directly. These dates also coincided with the creation of our institutions’ policies regarding administration of clarithromycin as adjunct surgical prophylaxis should azithromycin become unavailable for any reason. Although there have been studies that have demonstrated safety of clarithromycin use in pregnancy [29–31], clarithromycin had not been previously studied as adjunct surgical prophylaxis for non-elective cesarean deliveries.

It is important to note, however, that as the study was ongoing and more evidence for the effectiveness of treatments for COVID-19 were emerging, it was determined that the use of azithromycin with or without hydroxychloroquine was actually not effective in treating cases of confirmed SARS-CoV-2 [9–14]. This situation should prompt readers to maintain caution regarding the use of experimental drugs without high-quality evidence on a large scale in cases where little is known about an emerging disease.

## Conclusion

This study is the first to evaluate safety and effectiveness of clarithromycin as adjunctive antibiotic prophylaxis for patients undergoing non-elective cesarean delivery in comparison with no macrolides, to adapt to azithromycin shortages in COVID-19 pandemic. Our results demonstrate lower rates and decreased risk of development of postpartum endometritis when administering clarithromycin as adjunct surgical prophylaxis for non-elective cesarean deliveries as compared to no adjunct macrolide. These findings suggest that administration of clarithromycin for adjunctive surgical prophylaxis for non-elective cesarean deliveries may be a safe option that may provide suitable endometritis prophylaxis in cases where azithromycin is unavailable. However, given our study limitations as explained, a larger study would yield the most definitive conclusions on the effectiveness of clarithromycin as adjunct antibiotic prophylaxis for non-elective cesarean deliveries.

## Supporting information

S1 Data (PDF)

## References

[1] World Health Organization. Director-General's remarks at the media briefing on 2019-nCoV on 11 February 2020. https://www.who.int/dg/speeches/detail/who-director-general-s-remarks-at-the-media-briefing-on-2019-ncov-on-11-february-2020

[2] Holshue ML, DeBolt C, Lindquist S, Lofy KH, Wiesman J, Bruce H, et al First case of 2019 novel coronavirus in the United States. New England Journal of Medicine. 2020 1 31. 10.1056/NEJMoa2001191 PMC7092802 32004427

[3] Lane JC, Weaver J, Kostka K, Duarte-Salles T, Abrahao MT, Alghoul H, et al Safety of hydroxychloroquine, alone and in combination with azithromycin, in light of rapid wide-spread use for COVID-19: a multinational, network cohort and self-controlled case series study. medRxiv. 2020 1 1.

[4] Breslin N, Baptiste C, Miller R, Fuchs K, Goffman D, Gyamfi-Bannerman C, et al COVID-19 in pregnancy: early lessons. American Journal of Obstetrics & Gynecology MFM. 2020 3 27:100111 10.1016/j.ajogmf.2020.100111 32518902 PMC7271091

[5] Breslin N, Baptiste C, Gyamfi-Bannerman C, Miller R, Martinez R, Bernstein K, et al COVID-19 infection among asymptomatic and symptomatic pregnant women: Two weeks of confirmed presentations to an affiliated pair of New York City hospitals. American Journal of Obstetrics & Gynecology MFM. 2020 4 9:100118. 32292903 10.1016/j.ajogmf.2020.100118 PMC7144599

[6] Ai T, Yang Z, Hou H, Zhan C, Chen C, Lv W, et al Correlation of chest CT and RT-PCR testing in coronavirus disease 2019 (COVID-19) in China: a report of 1014 cases. Radiology. 2020 2 26:200642 10.1148/radiol.2020200642 32101510 PMC7233399

[7] Badreldin HA, Atallah B. Global drug shortages due to COVID-19: Impact on patient care and mitigation strategies. Research in Social and Administrative Pharmacy. 2020 5 19 10.1016/j.sapharm.2020.05.017 32446652 PMC7235598

[8] Catillon M, Majumder MS, Manzi SF, Mandl KD. News Coverage and Drug Shortages during the COVID-19 Pandemic. medRxiv. 2020 10 14.

[9] Cavalcanti AB, Zampieri FG, Azevedo LC, Rosa RG, Avezum A, Veiga VC, et al Hydroxychloroquine alone or in combination with azithromycin to prevent major clinical events in hospitalised patients with coronavirus infection (COVID-19): rationale and design of a randomised, controlled clinical trial. medRxiv. 2020 1 1.

[10] Gautret P, Lagier JC, Parola P, Meddeb L, Mailhe M, Doudier B, et al Hydroxychloroquine and azithromycin as a treatment of COVID-19: results of an open-label non-randomized clinical trial. International journal of antimicrobial agents. 2020 3 20:105949 10.1016/j.ijantimicag.2020.105949 32205204 PMC7102549

[11] Rosenberg ES, Dufort EM, Udo T, Wilberschied LA, Kumar J, Tesoriero J, et al Association of treatment with hydroxychloroquine or azithromycin with in-hospital mortality in patients with COVID-19 in New York state. Jama. 2020 5 11 10.1001/jama.2020.8630 32392282 PMC7215635

[12] Molina JM, Delaugerre C, Le Goff J, Mela-Lima B, Ponscarme D, Goldwirt L, et al No evidence of rapid antiviral clearance or clinical benefit with the combination of hydroxychloroquine and azithromycin in patients with severe COVID-19 infection. Med Mal Infect. 2020 3 30;50(384):30085–8. 10.1016/j.medmal.2020.03.006 32240719 PMC7195369

[13] Million M, Lagier JC, Gautret P, Colson P, Fournier PE, Amrane S, et al Full-length title: Early treatment of COVID-19 patients with hydroxychloroquine and azithromycin: A retrospective analysis of 1061 cases in Marseille, France. Travel medicine and infectious disease. 2020 5 5:101738 10.1016/j.tmaid.2020.101738 32387409 PMC7199729

[14] Furtado RH, Berwanger O, Fonseca HA, Corrêa TD, Ferraz LR, Lapa MG, et al Azithromycin in addition to standard of care versus standard of care alone in the treatment of patients admitted to the hospital with severe COVID-19 in Brazil (COALITION II): a randomised clinical trial. The Lancet. 2020 10 3;396(10256):959–67. 10.1016/S0140-6736(20)31862-6 32896292 PMC7836431

[15] Ohl CA, Dodds Ashley ES. Antimicrobial stewardship programs in community hospitals: the evidence base and case studies. Clinical infectious diseases. 2011 8 15;53(suppl_1):S23–8. 10.1093/cid/cir365 21795725

[16] Sturgill MG, Rapp RP. Clarithromycin: review of a new macrolide antibiotic with improved microbiologic spectrum and favorable pharmacokinetic and adverse effect profiles. Ann Pharmacother 1992; 26:1099 10.1177/106002809202600912 1421677

[17] Rapp RP, McCraney SA, Goodman NL, Shaddick DJ. New macrolide antibiotics: usefulness in infections caused by mycobacteria other than Mycobacterium tuberculosis. Ann Pharmacother 1994; 28:1255 10.1177/106002809402801109 7849341

[18] Tita AT, Szychowski JM, Boggess K, Saade G, Longo S, Clark E, et al Adjunctive azithromycin prophylaxis for cesarean delivery. N Engl J Med. 2016 9 29;375:1231–41. 10.1056/NEJMoa1602044 27682034 PMC5131636

[19] American College of Obstetricians and Gynecologists. ACOG practice bulletin number 47, October 2003: Prophylactic Antibiotics in Labor and Delivery. Obstetrics and gynecology. 2003 10;102(4):875 10.1016/s0029-7844(03)00984-0 14551023

[20] Franchi M, Raffaelli R, Baggio S, Scollo M, Garzon S, Laganà AS, et al Unintentional transvesical caesarean section: incidence, risk factors, surgical technique and post-operative management. European Journal of Obstetrics & Gynecology and Reproductive Biology. 2019 5 1;236:26–31. 10.1016/j.ejogrb.2019.02.023 30877907

[21] Häger RM, Daltveit AK, Hofoss D, Nilsen ST, Kolaas T, Øian P, et al Complications of cesarean deliveries: rates and risk factors. American journal of obstetrics and gynecology. 2004 2 1;190(2):428–34. 10.1016/j.ajog.2003.08.037 14981385

[22] Main EK, Chang SC, Cape V, Sakowski C, Smith H, Vasher J. Safety assessment of a large-scale improvement collaborative to reduce nulliparous cesarean delivery rates. Obstetrics & Gynecology. 2019 4 1;133(4):613–23. 10.1097/AOG.0000000000003109 30870288

[23] Macones GA, Caughey AB, Wood SL, Wrench IJ, Huang J, Norman M, et al Guidelines for postoperative care in cesarean delivery: Enhanced Recovery After Surgery (ERAS) Society recommendations (part 3). American journal of obstetrics and gynecology. 2019 9 1;221(3):247–e1. 10.1016/j.ajog.2019.04.012 30995461

[24] Welch BL. The significance of the difference between two means when the population variances are unequal. Biometrika. 1938 2 1;29(3/4):350–62.

[25] Yates F. Contingency tables involving small numbers and the χ 2 test. Supplement to the Journal of the Royal Statistical Society. 1934 1 1;1(2):217–35.Upton GJ. Fisher's exact test. J R Stat Soc Ser A Stat. 1992 Jan 1:395–402.

[26] Upton GJ. Fisher's exact test. Journal of the Royal Statistical Society: Series A (Statistics in Society). 1992 5;155(3):395–402.

[27] Zou G. A modified poisson regression approach to prospective studies with binary data. Am J Epidemiol. 2004 4 1;159(7):702–6. 10.1093/aje/kwh090 15033648

[28] Team, R. Core. "R: A language and environment for statistical computing." (2014).

[29] Einarson A, Phillips E, Mawji F, D'Alimonte D, Schick B, Addis A, et al A prospective controlled multicentre study of clarithromycin in pregnancy. American journal of perinatology. 1998;15(09):523–5. 10.1055/s-2007-994053 9890248

[30] Drinkard CR, Shatin D, Clouse J. Postmarketing surveillance of medications and pregnancy outcomes: clarithromycin and birth malformations. Pharmacoepidemiology and drug safety. 2000 12;9(7):549–56. 10.1002/pds.538 11338912

[31] Bar-Oz B, Diav-Citrin O, Shechtman S, Tellem R, Arnon J, Francetic I, et al Pregnancy outcome after gestational exposure to the new macrolides: a prospective multi-center observational study. European Journal of Obstetrics & Gynecology and Reproductive Biology. 2008 11 1;141(1):31–4. 10.1016/j.ejogrb.2008.07.008 18760873

