# Supplementary material for: Clarithromycin use for adjunct surgical prophylaxis before non-elective cesarean deliveries to adapt to azithromycin shortages in COVID-19 pandemic
Source: PLoS One. 2020 Dec 21;15(12):e0244266. doi: 10.1371/journal.pone.0244266 (PMC7751852; doi:10.1371/journal.pone.0244266)
Supplement: S1 Data — (PDF) [file pone.0244266.s001.pdf]

Deposit Agreement

This deposit agreement relates to the following digital data and associated metadata (hereinafter collectively referred to as "Dataset"):

Title: Clarithromycin use for adjunct surgical prophylaxis before non-elective cesarean deliveries to adapt to azithromycin shortages in COVID-19 pandemic
Persistent identifier: 10.17026/dans-xa2-awem
Deposit date: 2020-12-09

Parties

1. the organisation or person who is entitled to transfer and manage the Dataset, hereinafter referred to as *Depositor*,

Name: DJM Martingano
Organisation: St. John's Episcopal Hospital
Address: 327 Beach 19th Street
Postal code: 11691
Town: Far Rockaway
Country: USA
Telephone:
Email: danielmartinganodo@gmail.com

2. the organisation that is entitled to archive and manage the Dataset, hereinafter referred to as *Depositary*,

Organisation: Data Archiving and Networked Services (DANS), on behalf of the Royal Netherlands Academy of Arts and Sciences (KNAW)
Represented by: Dr. H. Wals, Director
Address: Anna van Saksenlaan 51
Postal code: 2593 HW
Town: The Hague
Country: The Netherlands
Telephone: +31 (0)70 349 4450
E-mail: info@dans.knaw.nl

DANS is an institute of KNAW (Royal Netherlands Academy of Arts and Sciences) and NWO (Dutch Research Council) with its registered office in The Hague.

DANS promotes sustainable access to digital research data. See www.dans.knaw.nl for more information.

Considering that:

The objective of the Depositary is to make research files permanently and digitally available;
The Depositary will only include and publish research files in its digital archival system if this can be done in a responsible manner;
The Depositor wishes to make use of the possibilities for digital archiving and accessibility of the deposited Dataset offered by the Depositary.

Agree as follows:

1. Deposit agreement

1. The Depositor will grant the Depositary a non-exclusive licence to the deposited digital data and associated metadata.
2. The Depositary will receive the right to include the Dataset in its digital archival system. The Depositary will transfer the contents of the Dataset to a compatible carrier in a manner and format of its choice.
3. Subject to the terms of this deposit agreement, the Depositary will receive the right to make the Dataset, or substantial parts of it, available to third parties by means of electronic distribution. In addition, the Depositary will have the right to make a copy of the Dataset, whether or not on behalf of third parties, or allow third parties to download a copy.
4. The licence will be granted free of charge. The Depositary will not charge costs for making the Dataset available.

2. Depositor

1. The Depositor declares to be the sole entitled party with regard to the intellectual property rights to the Dataset pursuant to, but not limited to, the Database Act, the Copyright Act and other relevant legislation, and/or to act with the permission of the titleholder(s) or co-titleholder(s).
2. The Depositor will indemnify the Depositary against all claims that others may make against the Depositary with regard to the Dataset or the deposit thereof, its format and/or contents or its availability for further research.
3. The Depositor confirms that the Dataset does not contain any data or other elements which, in isolation or upon disclosure outside the context of scientific research, are inconsistent with the Dutch Penal Code or other relevant national or international legislation.

3. Depositary

1. The Depositary will, to the best of its ability and resources, permanently archive the Dataset, preserving its readability and accessibility.
2. The Depositary will archive the Dataset unaltered and in its original software format as far as possible, taking into account the technological state of the art and the cost of implementation. The Depositary will have the right to change the design and/or functionality of the Dataset in so far as it is necessary to ensure the digital preservation, distribution or reusability of the Dataset.
3. If the Dataset has been assigned to the “Restricted Access” category, the Depositary will, to the best of its ability and resources, ensure effective technical provisions to prevent unauthorized third parties from accessing and/or consulting substantial parts of the Dataset.
4. The Depositor will remain the controller of the Dataset within the meaning of the General Data Protection Regulation (GDPR) insofar as the Dataset contains personal data within the meaning of the GDPR. The Depositary will be a processor within the meaning of the GDPR. If the Dataset contains personal data, the Depositor and the Depositary will conclude a processing agreement, except in the case of bibliographical data which exclusively refer to personal data that are necessary for the accountability of the Dataset, such as its creator, rights holders and citations (hereinafter: “Bibliographical Data”).

4. Dataset

1. The Dataset will consist of all the files transferred by the Depositor and the metadata provided by the Depositor. Metadata is understood to mean the contents of all fields that must be completed in the archival system at the time of deposit in order to describe the Dataset.
2. The Depositor declares that the Dataset corresponds to the metadata provided by the Depositor.

DANS promotes sustainable access to digital research data. See www.dans.knaw.nl for more information.

Data Archiving and Networked Services (DANS)
Anna van Saksenlaan 51 | 2593 HW Den Haag
070 349 44 50 | info@dans.knaw.nl | www.dans.knaw.nl
CoC 54667089 | DANS is an institute of KNAW and NWO

Driven by data

3. The Dataset shall be compiled with due observance of the Netherlands Code of Conduct for Research Integrity, the GDPR and other applicable laws and regulations.
4. The metadata and file names shall not contain any personal data within the meaning of the GDPR. Only Bibliographical Data are allowed. It is explicitly forbidden to include personal data that are part of the deposited data, such as - but not limited to - research subjects, in the metadata and file names.
5. The Depositor will provide the files in a preferred format, as defined on the Depositary's website at the time of deposit. In the event that a format is not defined as a preferred format, the Depositor will contact the Depositary before delivery. A different file format may only be supplied with the written consent of the Depositary.
6. The Depositor will provide documentation with the Dataset that explains its creation, contents and any specific values (such as codes, characters and abbreviations), its structure (such as folder structures and relationships between files) and its actual use (such as that of software) to third parties.
7. The Depositor will make the Dataset available to the Depositary in a manner and through a medium that the Depositary deems suitable.

5. Removing the Dataset and/or changing its accessibility

1. The Depositor may submit a reasoned request to the Depositary to make the Dataset temporarily or permanently unavailable to third parties or to remove it entirely or partly from the archival system. The Depositary will assess the request in view of its objective. In cases where the Dataset contains personal data, the Depositary will assess the request with due observance of the GDPR.
2. If there are compelling reasons to do so, the Depositary has the right to remove the Dataset, or part of it, from the archival system, or to limit or exclude access to it temporarily or permanently. In such cases, the Depositary will reasonably inform the Depositor.

6. Availability to third parties

1. All or part of the Dataset files will be made available to third parties in accordance with one or more access categories agreed with the Depositor: “Open Access” or “Restricted Access”, possibly with the addition of an embargo (see Appendix 1). If Restricted Access is applied, part of the files may be made available directly to third parties, in consultation with the Depositary.
2. If Open Access is applied, the Depositor determines whether the files in the Dataset are placed in the public domain or whether a licence (hereinafter: “Open Access Licence”) shall apply (see Appendix 1).
3. If the Open Access category has been agreed with the Depositor, the Depositary will make the Dataset available directly to third parties, providing either the public domain statement or the agreed Open Access Licence.
4. The Open Access Licence provided for the Open Access category by the Depositor will apply to all files and their contents, to the extent that they are protected by copyright. The Depositary will make the Dataset as a whole, as well as its content or parts of its content, available under the conditions of the specified licence.
5. If the files in the Dataset, or parts of them, contain personal data within the meaning of the GDPR, the Restricted Access category will be applied exclusively and the files will not be made available directly to third parties.
6. If the Depositor and Depositary agree on Restricted Access, the following shall apply:
    1. The Depositary will make the files in the Dataset, or parts of them, exclusively available to users registered with the Depositary with whom they have agreed the DANS Licence (see Appendix 2) (hereinafter: “Users”). The Depositor has taken note of the DANS Licence and agrees to make the Dataset available in accordance with this licence. The Depositor is free to impose further conditions on Users prior to making the Dataset available.
    2. In addition to the aforementioned provisions, the Depositary will only make the files in the Dataset, or parts of them, available to Users who have submitted a permission request for access to the Dataset and who have subsequently been granted such permission by the Depositor.
    3. If a Dataset with Restricted Access includes files which the Depositor wishes to provide without the required permission as referred to under (b), the Depositary shall make the files directly available to third parties at the request of the Depositor, referring to the DANS Licence.
    4. The Depositor will ensure the availability of a stable email address and check it regularly so that permission requests can be processed within a reasonable period of time.

DANS promotes sustainable access to digital research data. See www.dans.knaw.nl for more information.

Data Archiving and Networked Services (DANS)
Anna van Saksenlaan 51 | 2593 HW Den Haag
070 349 44 50 | info@dans.knaw.nl | www.dans.knaw.nl
CoC 54667089 | DANS is an institute of KNAW and NWO

Driven by data

    5. The Depositary will facilitate consultations between the Depositor and the User who wants to use a Dataset, but cannot be held responsible for the Depositor's decision whether or not to make the Dataset available, nor for any conditions under which this is done.
7. Contrary to the above, the Depositary may make the Dataset or substantial parts of it available to third parties:
    1. if the Depositary is obliged to do so by virtue of laws and regulations, a judicial decision, or by a supervisory body;
    2. if this is necessary for preserving the Dataset or the archival system;
    3. if the Depositary ceases to exist and/or terminates its activities in the field of data archiving, or transfers its activities to a similar institution in compliance with Article 8.
8. The Depositary will make the metadata associated with the Dataset freely available.
9. The metadata associated with the Dataset will be included in the Depositary's databases and publications, and will be accessible to everyone.
10. When a Dataset no longer contains any personal data within the meaning of the GDPR, the Depositor and the Depositary may decide to change its access category.

7. Using Datasets with Restricted Access

1. The Depositary will oblige Users to whom it makes a Dataset or substantial parts of it available to include in their research results an unambiguous acknowledgement of the source, as specified in the DANS Licence, regarding the Dataset whose data has been used.
2. The Depositary will oblige Users to whom it makes the Dataset available to respect any copyright and database rights pertaining to the Dataset, as indicated by the Depositor.
3. In the event that the Depositary finds a violation, the Depositary will contact the Depositor and the User will be excluded from further use of Datasets until the issue has been resolved with the Depositor.
4. The Depositor will notify the Depositary as soon as possible of a failure to cite the source or an infringement of copyright or database rights as referred to in this article, to allow the Depositary to take the measures that it deems necessary, including, but not limited to, exclusion from further use of Datasets.

8. Death of the Depositor or liquidation of its organisation

Upon the death of the Depositor or the liquidation or termination of its organisation, if no legal successors or co-entitled parties are known to the Depositary, the Depositary will be entitled to do whatever it deems reasonably necessary with a view to achieving its objective. In the event that the Dataset contains personal data within the meaning of the GDPR, the Depositary will request another organisation to act as the Depositor. This request will only be made to an organisation established in the Netherlands that, according to its statutes or by law, has scientific research, or the support or promotion of it, among its core tasks and/or objectives.

9. Liability

1. Barring intent and/or gross negligence, the Depositor will indemnify the Depositary against all liability resulting from complete or partial loss of the Dataset.
2. Barring intent and/or gross negligence, the Depositor will indemnify the Depositary against all claims from third parties that may be brought against the Depositary as a result of the Dataset being lost in whole or in part, as well as against the costs that the Depositary will incur, for example for legal proceedings.

10. Duration, cancellation, termination of the agreement

1. This agreement takes effect on the date of the acceptance of this deposit agreement by the Depositor. The Depositary will then publish the Dataset as soon as possible.
2. This agreement will remain in effect for an indefinite period of time, unless:
    1. Either of the parties cancels the agreement in writing, subject to a notice period of six months;
    2. Pursuant to Article 5 of this agreement, the Dataset has been permanently removed from the archival system;
    3. Article 8 applies.
3. Changing the access category or Open Access Licence is always permitted at the written request of the Depositor, with due observance of Article 6(5) of this agreement.

Data Archiving and Networked Services (DANS)
Anna van Saksenlaan 51 | 2593 HW Den Haag
070 349 44 50 | info@dans.knaw.nl | www.dans.knaw.nl
CoC 54667089 | DANS is an institute of KNAW and NWO

11. Applicable law

1. This agreement is governed by Dutch law.
2. Disputes that cannot be resolved amicably will be submitted to the competent court in the Amsterdam district.

This agreement has been accepted by both parties on 2020-12-09 upon completion of the deposit process via easy.dans.knaw.nl.

DANS promotes sustainable access to digital research data. See www.dans.knaw.nl for more information.

Data Archiving and Networked Services (DANS)
Anna van Saksenlaan 51 | 2593 HW Den Haag
070 349 44 50 | info@dans.knaw.nl | www.dans.knaw.nl
CoC 54667089 | DANS is an institute of KNAW and NWO

Appendix 1 Dataset availability

The Dataset will be distributed and made available by the Depositary in the manner set out below. If the Dataset contains personal data within the meaning of the GDPR, with the exception of Bibliographical Data, the only access category permitted is Restricted Access. Metadata will always be made freely available.

Open Access

The files in the Dataset will be directly accessible to third parties. Third parties do not have to register with the Depositary. The Dataset will be placed in the public domain or made available under an Open Access Licence.

Using the following public-domain statement or Open Access Licence:

CC0-1.0 : http://creativecommons.org/publicdomain/zero/1.0 (see Appendix 3)

DANS promotes sustainable access to digital research data. See www.dans.knaw.nl for more information.

Data Archiving and Networked Services (DANS)
Anna van Saksenlaan 51 | 2593 HW Den Haag
070 349 44 50 | info@dans.knaw.nl | www.dans.knaw.nl
CoC 54667089 | DANS is an institute of KNAW and NWO

Driven by data

Appendix 2 DANS Licence

DANS Licence

Effective from 9 January 2020

This licence sets out the conditions for using datasets to which the access categories “Open access for registered users” and “Restricted access” apply.

Anyone to whom DANS, on behalf of the holder of the rights to the dataset, makes one or more files of a dataset available (hereinafter referred to as the “User”), agrees to the following conditions. Acceptance of the conditions establishes an agreement between DANS and the User.

1. Responsible use

The User will act in accordance with the Netherlands Code of Conduct for Research Integrity, the GDPR and other applicable laws and regulations.

2. Citing the dataset

The User will always cite the dataset in the research results they publish, in whatever form, when it has been used in the research.

This source reference will at least consist of:

- The names and/or organisations of the producers of the dataset;
- The year in which the dataset was produced;
- The title of the dataset;
- The name of organisation managing the archive in which the dataset is stored: DANS;
- The persistent identifier of the dataset as a full URL.

For example:

Doorn, dr P.K.; Bommeljé, drs L.S.; Vroom, dr J.A.C.; Wijngaarden, drs H. van;
Bommeljé, drs Y.B. (1990): *The Aetolian Studies Project*.
DANS. https://doi.org/10.17026/dans-xxu-6utq

3. Distribution or disclosure of the dataset

The User shall respect all intellectual property rights to the dataset, such as copyrights, database and/or neighbouring rights.

For distribution or disclosure of the entire dataset or of substantial parts thereof, the User must first request permission from the holder of the rights to the dataset. This is the person(s) and/or institution(s) listed in the “Rights holder” metadata field of the dataset. If no holder is listed in this field, the User must contact the person(s) and/or organisation of the person(s) who produced the dataset.

4. Statement when distributing or disclosing the dataset

When distributing or disclosing the entire dataset or substantial parts of it, in the manner described in Article 2 of this DANS Licence and with the permission of the rights holder, the User shall, in addition to the acknowledgement referred to in Article 2, declare at all times:

- the name of the dataset rights holder;

DANS promotes sustainable access to digital research data. See www.dans.knaw.nl for more information.

Data Archiving and Networked Services (DANS)
Anna van Saksenlaan 51 | 2593 HW Den Haag
070 349 44 50 | info@dans.knaw.nl | www.dans.knaw.nl
CoC 54667089 | DANS is an institute of KNAW and NWO

Driven by data

- that this rights holder has granted permission for the distribution;
- that further distribution by third parties is not permitted without the consent of the dataset rights holder.

5. Publications

The User will inform DANS of the publications for which the dataset has been used. In this context, publications are defined as publications with an internationally recognized standard identification number, such as ISBN, ISSN or DOI. If a publication is available on the internet, the User will pass on the URL to DANS. If a publication is not available on the internet (via an URL), the User will pass on the source reference to DANS.

6. Personal data

The User will always be responsible for the processing of personal data made available within the meaning of the GDPR and any other relevant privacy legislation, as well as for complying with any conditions set by the depositor.

7. Liability for content

DANS shall in no way be liable for the contents or accompanying documentation of the dataset, including infringements of privacy rights within the meaning of the GDPR, unless in the event of intent or gross negligence on the part of DANS. The User is requested to inform DANS of any inaccuracies found as soon as possible after their discovery.

Neither DANS nor the depositor provide any guarantee that a dataset made available will meet the research objectives of the User. Neither DANS nor the depositor are liable for conclusions based on the dataset.

8. Non-compliance with licence conditions

a. If the licence conditions are not complied with, the use of the dataset must immediately be discontinued upon DANS's first request. DANS reserves the right to inform the User's employer. In the event of unlawful use of personal data, DANS has the right to inform the Data Protection Authority as well. These measures are without prejudice to the authority of DANS to hold the User to account in court in the event of non-compliance or insufficient compliance with this licence.
b. If the licence conditions are not complied with, the User's access to datasets other than that of the rights holder will be suspended until the issue has been resolved in consultation with the User, their employer and the rights holder (where applicable).
c. The User will indemnify DANS against all claims by third parties which are directly or indirectly related to the use of a dataset made available.

9. Compelling reasons

For compelling reasons, such as, but not limited to, an infringement of other people's copyright or an infringement of the Code of Conduct for Research Integrity, DANS has the right to order the User to stop using the dataset.

10. Changes to the agreement

DANS reserves the right to unilaterally change this agreement. In the event of substantial changes, DANS will inform the User, through EASY or by other means, before the new conditions take effect, so that the User has the opportunity to become aware of the changes. If the User does not accept the changes, the User must stop using the dataset(s) and delete any downloaded files. By continuing to use the dataset(s) after the changes have taken effect, the User accepts the updated conditions.

Data Archiving and Networked Services (DANS)
Anna van Saksenlaan 51 | 2593 HW Den Haag
070 349 44 50 | info@dans.knaw.nl | www.dans.knaw.nl
CoC 54667089 | DANS is an institute of KNAW and NWO

11. Applicable law

a. This licence is governed by Dutch law.
b. Disputes that cannot be resolved amicably will be submitted to the competent court in the Amsterdam district.

DANS promotes sustainable access to digital research data. See www.dans.knaw.nl for more information.

Data Archiving and Networked Services (DANS)
Anna van Saksenlaan 51 | 2593 HW Den Haag
070 349 44 50 | info@dans.knaw.nl | www.dans.knaw.nl
CoC 54667089 | DANS is an institute of KNAW and NWO

Appendix 3 Legal text of chosen public-domain statement or Open Access Licence

```
Creative Commons Legal Code

Statement of Purpose

The laws of most jurisdictions throughout the world
automatically confer exclusive Copyright and Related Rights
(defined below) upon the creator and subsequent owner(s) (each
and all, an "owner") of an original work of authorship and/or
a database (each, a "Work").

Certain owners wish to permanently relinquish those rights
to a Work for the purpose of contributing to a commons of
creative, cultural and scientific works ("Commons") that the
public can reliably and without fear of later claims of
infringement build upon, modify, incorporate in other works,
reuse and redistribute as freely as possible in any form
whatsoever and for any purposes, including without limitation
commercial purposes. These owners may contribute to the
Commons to promote the ideal of a free culture and the further
production of creative, cultural and scientific works, or to
gain reputation or greater distribution for their Work in part
through the use and efforts of others.

For these and/or other purposes and motivations, and
without any expectation of additional consideration or
compensation, the person associating CC0 with a Work (the
"Affirmer"), to the extent that he or she is an owner of
Copyright and Related Rights in the Work, voluntarily elects
to apply CC0 to the Work and publicly distribute the Work
under its terms, with knowledge of his or her Copyright and
Related Rights in the Work and the meaning and intended legal
effect of CC0 on those rights.

1. Copyright and Related Rights.
A Work made available under CC0 may be protected by
copyright and related or neighboring rights ("Copyright and
Related Rights"). Copyright and Related Rights include, but
are not limited to, the following:


  i.    the right to reproduce, adapt, distribute, perform,
        display, communicate, and translate a Work;
  ii.   moral rights retained by the original author(s) and/or
        performer(s);
  iii.  publicity and privacy rights pertaining to a person's
        image or likeness depicted in a Work;
  iv.   rights protecting against unfair competition in regards
        to a Work, subject to the limitations in paragraph 4(a),
        below;
  v.    rights protecting the extraction, dissemination, use and
        reuse of data in a Work;
  vi.   database rights (such as those arising under Directive
        96/9/EC of the European Parliament and of the Council of 11
        March 1996 on the legal protection of databases, and under
        any national implementation thereof, including any amended
        or successor version of such directive); and
  vii.  other similar, equivalent or corresponding rights
        throughout the world based on applicable law or treaty, and
        any national implementations thereof.


2. Waiver. To the greatest extent
 permitted by, but not in contravention of, applicable law,
 Affirmer hereby overtly, fully, permanently, irrevocably and
 unconditionally waives, abandons, and surrenders all of
 Affirmer's Copyright and Related Rights and associated claims
```


DANS promotes sustainable access to digital research data. See www.dans.knaw.nl for more information.

Data Archiving and Networked Services (DANS)
Anna van Saksenlaan 51 | 2593 HW Den Haag
070 349 44 50 | info@dans.knaw.nl | www.dans.knaw.nl
CoC 54667089 | DANS is an institute of KNAW and NWO

Driven by data

and causes of action, whether now known or unknown (including existing as well as future claims and causes of action), in the Work (i) in all territories worldwide, (ii) for the maximum duration provided by applicable law or treaty (including future time extensions), (iii) in any current or future medium and for any number of copies, and (iv) for any purpose whatsoever, including without limitation commercial, advertising or promotional purposes (the "Waiver"). Affirmer makes the Waiver for the benefit of each member of the public at large and to the detriment of Affirmer's heirs and successors, fully intending that such Waiver shall not be subject to revocation, rescission, cancellation, termination, or any other legal or equitable action to disrupt the quiet enjoyment of the Work by the public as contemplated by Affirmer's express Statement of Purpose.

3. Public License Fallback. Should any part of the Waiver for any reason be judged legally invalid or ineffective under applicable law, then the Waiver shall be preserved to the maximum extent permitted taking into account Affirmer's express Statement of Purpose. In addition, to the extent the Waiver is so judged Affirmer hereby grants to each affected person a royalty-free, non transferable, non sublicensable, non exclusive, irrevocable and unconditional license to exercise Affirmer's Copyright and Related Rights in the Work (i) in all territories worldwide, (ii) for the maximum duration provided by applicable law or treaty (including future time extensions), (iii) in any current or future medium and for any number of copies, and (iv) for any purpose whatsoever, including without limitation commercial, advertising or promotional purposes (the "License"). The License shall be deemed effective as of the date CC0 was applied by Affirmer to the Work. Should any part of the License for any reason be judged legally invalid or ineffective under applicable law, such partial invalidity or ineffectiveness shall not invalidate the remainder of the License, and in such case Affirmer hereby affirms that he or she will not (i) exercise any of his or her remaining Copyright and Related Rights in the Work or (ii) assert any associated claims and causes of action with respect to the Work, in either case contrary to Affirmer's express Statement of Purpose.

4. Limitations and Disclaimers.

a. No trademark or patent rights held by Affirmer are waived, abandoned, surrendered, licensed or otherwise affected by this document.
b. Affirmer offers the Work as-is and makes no representations or warranties of any kind concerning the Work, express, implied, statutory or otherwise, including without limitation warranties of title, merchantability, fitness for a particular purpose, non infringement, or the absence of latent or other defects, accuracy, or the present or absence of errors, whether or not discoverable, all to the greatest extent permissible under applicable law.
c. Affirmer disclaims responsibility for clearing rights of other persons that may apply to the Work or any use thereof, including without limitation any person's Copyright and Related Rights in the Work. Further, Affirmer disclaims responsibility for obtaining any necessary consents, permissions or other rights required for any use of the Work.
d. Affirmer understands and acknowledges that Creative Commons is not a party to this document and has no duty or obligation with respect to this CC0 or use of the Work.

Data Archiving and Networked Services (DANS)
Anna van Saksenlaan 51 | 2593 HW Den Haag
070 349 44 50 | info@dans.knaw.nl | www.dans.knaw.nl
CoC 54667089 | DANS is an institute of KNAW and NWO
